# Three-Dimensional Ti_3_C_2_T_*x*_ MXene-Prussian Blue Hybrid Microsupercapacitors by Water Lift-Off Lithography

**DOI:** 10.1021/acsnano.1c06552

**Published:** 2022-01-28

**Authors:** Yongjiu Lei, Wenli Zhao, Yunpei Zhu, Ulrich Buttner, Xiaochen Dong, Husam N. Alshareef

**Affiliations:** ^†^Materials Science and Engineering, Physical Science and Engineering Division and ^§^Nanofabrication Core Lab, King Abdullah University of Science and Technology (KAUST), Thuwal 23955-6900, Saudi Arabia; ‡ School of Physical and Mathematical Sciences, Nanjing Tech University, Nanjing 211816, China

**Keywords:** Ti_3_C_2_T_*x*_ MXene, Prussian blue, hybrid microsupercapacitors, water lift-off lithography, laser printing

## Abstract

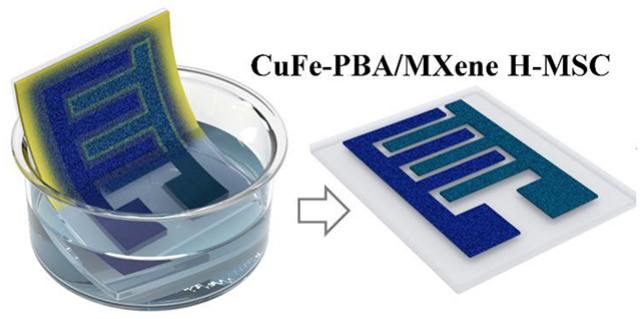

The construction of electrochemical energy-storage devices by scalable thin-film microfabrication methods with high energy and power density is urgently needed for many emerging applications. Herein, we demonstrate an in-plane hybrid microsupercapacitor with a high areal energy density by employing a battery-type CuFe-Prussian blue analogue (CuFe-PBA) as the positive electrode and pseudocapacitive titanium carbide MXene (Ti_3_C_2_T_*x*_) as the negative electrode. A three-dimensional lignin-derived laser-induced graphene electrode was prepared as the substrate by laser exposure combined with an environmentally friendly water lift-off lithography. The designed hybrid device achieved enhanced electrochemical performance thanks to the ideal match of the two types of high-rate performance materials in proton-based electrolytes and the numerous electrochemically active sites. In particular, the device delivers a high areal capacitance of 198 mF cm^–2^, a wide potential window (1.6 V), an ultrahigh rate performance (75.8 mF cm^–2^ retained even at a practical/high current density of 100 mA cm^–2^), and a competitive energy density of 70.5 and 27.6 μWh cm^–2^ at the power densities 0.74 and 52 mW cm^–2^, respectively. These results show that the Ti_3_C_2_T_*x*_/CuFe-PBA hybrid microsupercapacitors are promising energy storage devices in miniaturized portable and wireless applications.

The accelerating growth of the Internet of things (IoT), especially the development of miniaturized portable and wearable electronic devices, is a significant emerging application for compatible microscale power systems.^1,2^ It is highly desirable to develop efficient, miniaturized, and integrable energy storage modules with rapid and continuous power delivery.^3,4^ Microbatteries (MBs) possess a high energy density, but they fail to meet the fast charge–discharge rate and long cycling life requirement at this stage.^2,5^ As an alternative to MBs, microsupercapacitors (MSCs) hold great promise for high-power delivery, fast rate capability, and extended lifetime microdevices to support the IoT’s rapid development.^5−9^

To date, the interdigitated architecture is the most commonly applied in-plane design of MSCs.^10^ Finger electrodes are fabricated on the same plane as the current collectors and electronically separated by an inactive gap. A solid-state or gel-type electrolyte is coated on the top to ensure ion transport along the basal plane of the electrodes. The in-plane interdigital finger structure of MSCs offers several advantages over the conventional sandwich structure but loses in areal performance.^11^ One way to increase the areal performance of MSCs is to improve the mass loading of the active electrode materials and retain the high-rate performance of the MSCs at the same time. Porous three-dimensional (3D) planar interdigitated MSCs expose surfaces of electrodes in all three dimensions and ensure a large specific electrode surface area that could be an effective method for interdigitated MSCs fabrication. As an example, an extrusion-based 3D printing technique has been used to fabricate 3D porous MSCs with good electrochemical performance.^12,13^ Recently, a laser-based printing technique has emerged as an effective and reliable method of constructing miniaturized systems with high printing resolution.^14−16^ It allows various materials to be used, including polymers, metals, and ceramics, and does not need complicated printable inks compared to extrusion-based 3D printing methods.^17^ Laser-induced graphene (LIG) is a graphitic carbon with a 3D structure that is formed when various substrates are exposed to laser irradiation. The LIG electrode, sometimes also called laser-scribed graphene, has a high specific surface area with a honeycomb architecture composed of a porous, mechanically reinforced framework that facilitates electron transport, ionic diffusion, thus simultaneously ensuring high capacitance and high power density.^18,19^

In recent years, Ti_3_C_2_T_*x*_ MXene has gained considerable interest among the supercapacitor and battery communities because of its metallic conductivity (typically 8000 S cm^–1^, but can be higher) and surprising stability in aqueous dispersions and redox reactions on the surface layers of titanium atoms.^20,21^ These features have led to high pseudocapacitance, high-rate performance, and long-term cycling stability.^22,23^ On the other hand, Prussian blue analog (PBA) has been used as battery electrode material due to several advantages: (i) the open framework inside the crystals, which makes it easier for the diffusion of charge carrier ions, ensuring a high-rate performance; (ii) the high framework stability results in a long cycle life; and (iii) PBA materials are inexpensive and thus are suitable for large-scale applications.^24^ Among various PBA materials, the Cu[Fe(CN)_6_]_0.63_δ_0.37_·3.4H_2_O (CuFe-PBA), wherein δ means the ferricyanide vacancy, displays outstanding electrochemical performance, such as superior proton conduction, ultrahigh rate performance, and extremely long cycle life.^25^ These key features make the CuFe-PBA closely matched for use as a high-rate battery material with Ti_3_C_2_T_*x*_ to assemble hybrid microsupercapacitors (H-MSCs). Ti_3_C_2_T_*x*_ MXene and CuFe-PBA are a good electrode pair for H-MSCs because: (i) their operating voltage windows are complementary with a positive window for CuFe-PBA and negative window for Ti_3_C_2_T_*x*_, which maximizes the potential working window to increase the energy density of the device; and (ii) both CuFe-PBA and Ti_3_C_2_T_*x*_ can display high-rate electrochemical performance in common acidic electrolytes.

Herein, we demonstrate an H-MSCs with an enhanced electrochemical performance by pairing the CuFe-PBA as the battery-like positive electrode with Ti_3_C_2_T_*x*_ as the capacitive negative electrode on a 3D lignin-derived LIG electrode using a green water-based lift-off lithography method. The hierarchical porous architecture of the LIG allows a higher mass loading of the active materials, larger contact surface area, and faster ion diffusion, which can enhance the areal power and energy density. The H-MSCs deliver a high specific capacitance of 198 mF cm^–2^ and a wide working voltage of 1.6 V. Ti_3_C_2_T_*x*_-based symmetric MSCs were prepared based on the lignin-based LIG electrode by a mask-free and green water-based lift-off lithography method. Series-connected MSCs with a wide voltage window of 9 V were also fabricated, which delivers a competitive energy density of 34 μWh cm^–2^ at the power densities of 21 mW cm^–2^. This work successfully demonstrates the good performance of MXene-based hybrid microsupercapacitors by scalable microfabrication methods toward integrated micropower units in future portable and wireless devices.

## Results and Discussion

### Negative Electrode Design and Fabrication

**Figure 1:**
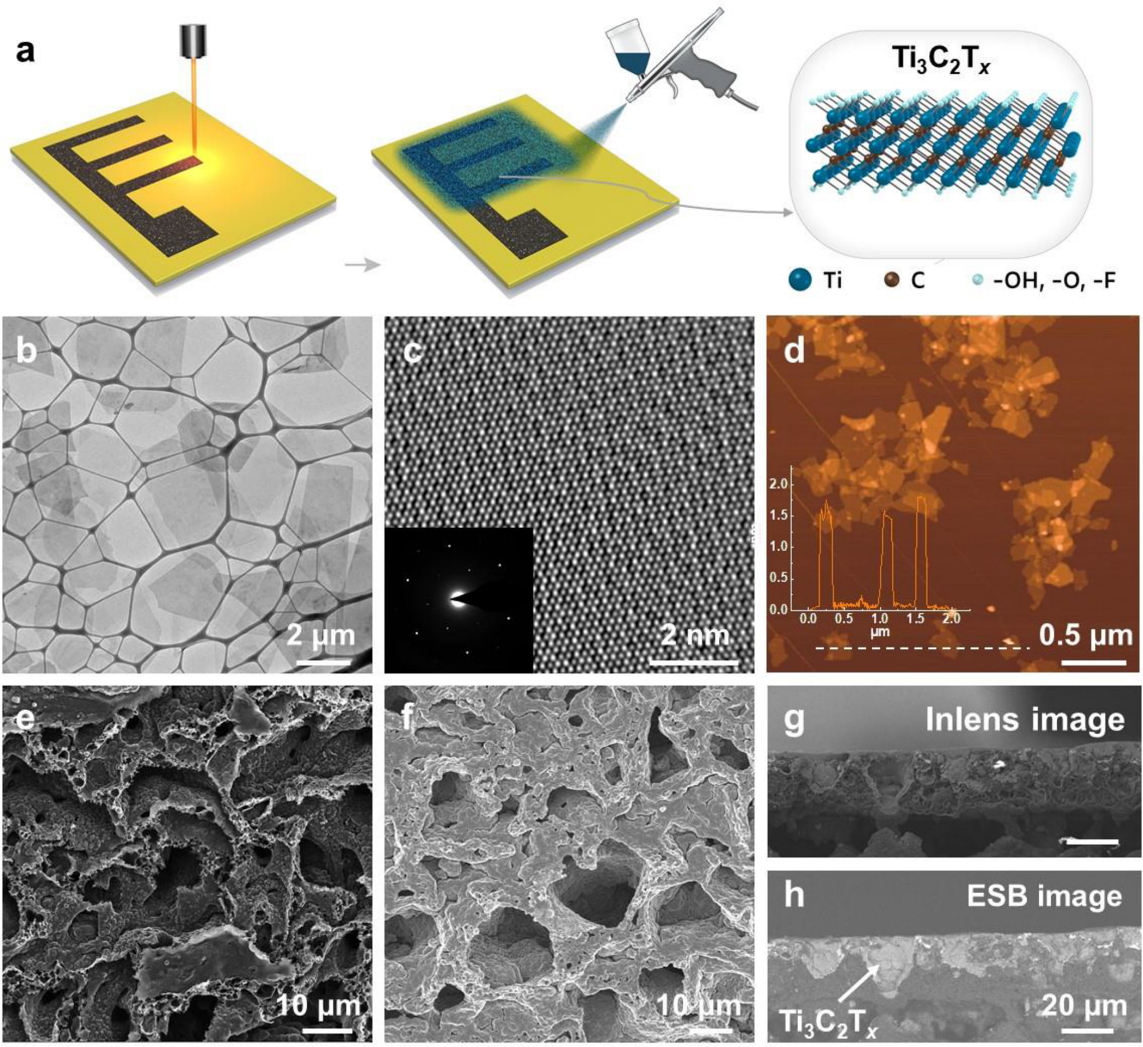
(a) Schematic illustration of the fabrication process of Ti_3_C_2_T_*x*_/LIG electrode. (b) TEM image of the delaminated Ti_3_C_2_T_*x*_ flakes. (c) HRTEM of the Ti_3_C_2_T_*x*_ nanosheet, inset is the SAED pattern of the Ti_3_C_2_T_*x*_ nanosheet. (d) AFM image of the Ti_3_C_2_T_*x*_ flake after sonication; insert figure is the height profile associated with the crossed line. (e) SEM image of the LIG electrode shows 3D porous structure. (f) SEM image of the Ti_3_C_2_T_*x*_/LIG electrode after the spray coating process. (g) Inlens SEM image of the side view of the Ti_3_C_2_T_*x*_/LIG electrode. (h) ESB SEM image of the side view of the Ti_3_C_2_T_*x*_/LIG electrode.

The fabrication process of the Ti_3_C_2_T_*x*_/LIG electrode is presented in Figure 1a using a simple mask-free spray coating method. First, the 3D porous LIG electrode pattern was fabricated using a CO_2_ laser (as shown in Experimental Section). A poly(vinyl alcohol) (PVA)/lignin film on the polymer substrate was prepared by a blade-coating method using the PVA/lignin ink (10 wt % in water), as shown in Figure S1. After natural drying, the as-prepared film was transformed into 3D porous LIG electrodes with designed patterns by a CO_2_ laser. The water lift-off process then uses the high water solubility of alkaline lignin and PVA, removing the parts of the lignin/PVA film that were unexposed to the CO_2_ laser and leaving the transformed LIG electrode on the surface of the substrate. This process developed by our group and known as lignin-laser lithography (LLL) is shown in Video S1 and Figure S1.^43^ During the process, lignin, which is recycled from waste, is transformed into high-value-added graphene electrodes. The patterned LIG electrode was subject to various characterization techniques, as shown in Figure S2. The 3D porous morphology of LIG was validated by scanning electron microscopy (SEM) and transmission electron microscopy (TEM). The SEM image in Figures 1d and S2c and the TEM image in Figure S2a show the 3D interconnected architecture nature of LIG, which favors the electron transport and ions diffusion. Figure S14 shows the adsorption and desorption isotherms of N_2_ at 77 K of the 3D LIG electrode material, revealing that the LIG electrode is a microporous material. A high-resolution TEM (HRTEM) image (Figure S2b) shows that the multilayer graphene’s interplanar distance is about 0.349 nm, which matches the previous reports.^18^ Raman spectra of the LIG substrate have three prominent peaks (Figure S2d): D peak (the number of defects/functional groups) at 1360 cm^–1^, and G peak (comes from the E_2g_ phonons of C sp^2^ atoms) at 1570 cm^–1^ and 2D peak (corresponding to second-order zone-boundary phonons) at 2700 cm^–1^.^26^ The weak D peak, strong G peak, and low *I*_D_/*I*_G_ ratio (0.36) reveal a well-defined graphene framework.^27^

For microfabrication of the Ti_3_C_2_T_*x*_/LIG electrode, a hydrophilic Ti_3_C_2_T_*x*_ flakes dispersion was first prepared by the minimal intensive layer delamination (MILD) strategy (see the synthesis details in Experimental Section).^20^ The synthesized Ti_3_C_2_T_*x*_ dispersions exhibit strong colloidal stability (approved by the Tyndall scattering effect, Figure S3a), enabling a 3D porous Ti_3_C_2_T_*x*_/LIG electrode by a spray coating process without a surfactant additive. The delaminated Ti_3_C_2_T_*x*_ flakes (Figure 1b) show well-structured 2D nanosheet morphology, with an average flake size of around 3 μm. Moreover, the HRTEM image (Figure 1c) shows Ti and C atoms’ hexagonal arrangement inherited from Ti_3_AlC_2_, indicating removal of the Al layers (which is also confirmed by the X-ray diffractometer patterns in Figure S4a). The associated selected area electron diffraction (SAED) pattern (inset of Figure 1c) shows hexagonal arrangement spots, indicating the single-crystal nature of the Ti_3_C_2_T_*x*_ nanosheet.^28^

The large-sized Ti_3_C_2_T_*x*_ flakes dispersion was broken into small pieces by a tip sonicator to make it contact well with the porous LIG electrode. The atomic force microscopy (AFM) image (Figure 1d) indicates that the lateral size of Ti_3_C_2_T_*x*_ flakes after sonication ranges from 50 to 500 nm, giving an average size of about 220 nm (Figure S3b). The small-sized Ti_3_C_2_T_*x*_ nanosheets show about 1.5 nm in thickness, confirming that Ti_3_C_2_T_*x*_ MXene is fully delaminated. Figure 1f shows the SEM image of the Ti_3_C_2_T_*x*_/LIG electrode, prepared by spraying the small-sized Ti_3_C_2_T_*x*_ flakes dispersion on the LIG electrode. The 3D hierarchical porous architecture maintains well after the spray coating process. Furthermore, the cross-section morphology of the Ti_3_C_2_T_*x*_/LIG electrode was investigated by SEM combined with an energy selective backscattered (ESB) detector to image clear composition.^29^ As shown in Figure 1g,h, the Ti_3_C_2_T_*x*_ MXene nanosheets were homogeneously coated on the scaffold surface of 3D LIG.

**Figure 2:**
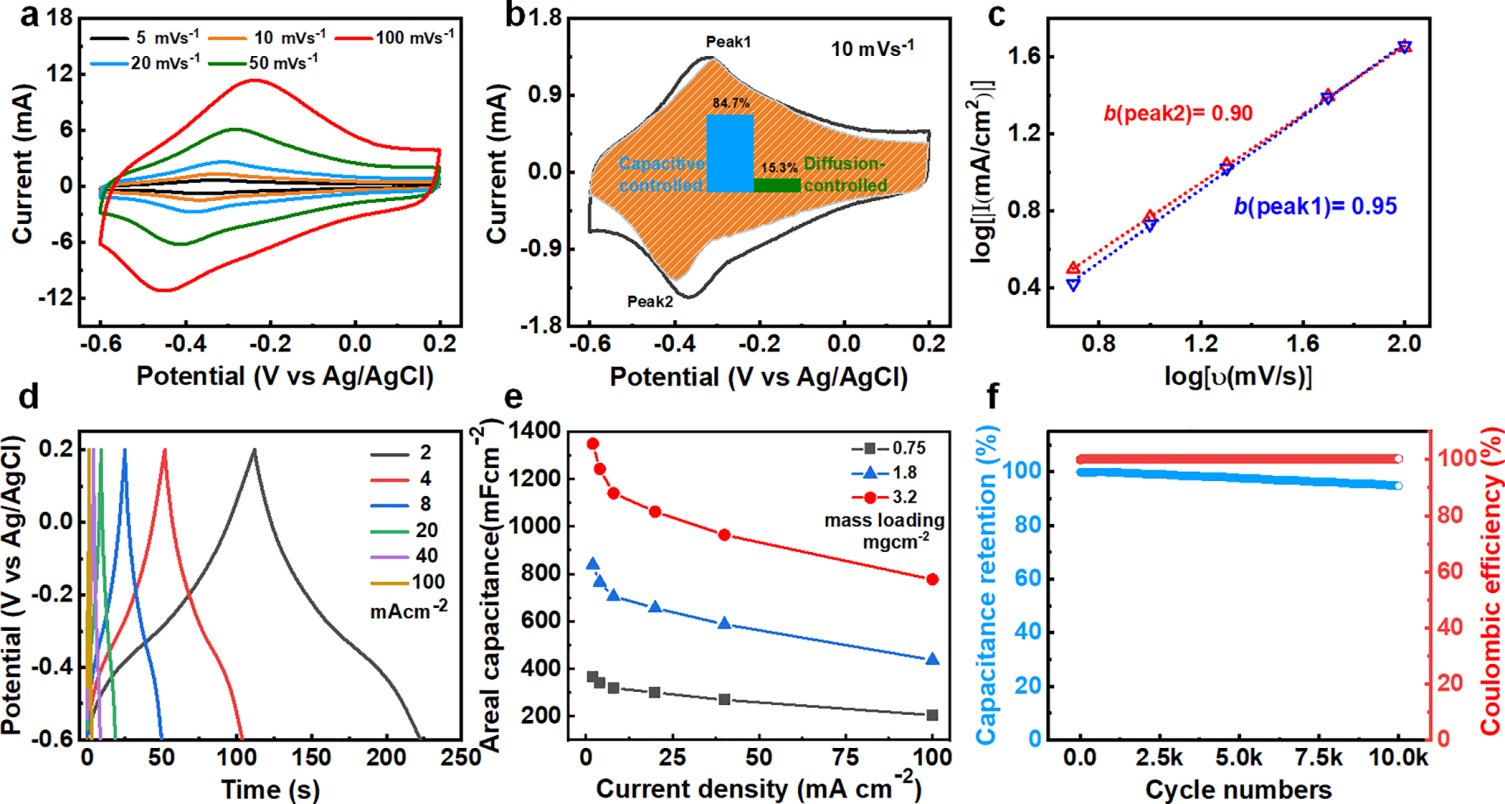
(a) CV curves of Ti_3_C_2_T_*x*_/LIG electrode at the scan rates of 5–100 mV s^–1^ in the potential range of −0.6 to 0.2 V (*vs* Ag/AgCl). (b) Deconvolution of charge storage contributions of the Ti_3_C_2_T_*x*_/LIG electrode (capacitive-controlled vs diffusion-controlled currents). (c) The *b* values derived from the CV curves according to eq 1. (d) GCD profiles of the Ti_3_C_2_T_*x*_/LIG electrode at the current density of 2–100 mA cm^–2^. (e) Areal capacitance calculated from the GCD profiles. (f) The cycling performance of the Ti_3_C_2_T_*x*_/LIG electrode at the fixed current density of 20 mA cm^–2^.

The electrochemical performance of the Ti_3_C_2_T_*x*_/LIG electrode was first evaluated in a three-electrode system in 2 M H_2_SO_4_. To identify a suitable operating potential window, cyclic voltammogram (CV) curves of Ti_3_C_2_T_*x*_/LIG electrode (mass loading of 0.75 mg cm^–2^) were collected with varied scan rates (Figure 2a). Two broad redox peaks have appeared in the potential range of −0.6 to 0.2 V (*vs* Ag/AgCl). The peaks slightly shift (anodic: −0.33 V to −0.24 V, cathodic: −0.35 V to −0.45 V) when increasing the scan rate from 5 mV/s to 50 mV/s, indicating a reversible redox reaction has occurred on the surface of the Ti_3_C_2_T_*x*_ MXene. The equivalent series resistance (ESR) collected from the Ti_3_C_2_T_*x*_/LIG electrode was 2.3 Ω, and the very low resistance confirmed the good conductivity of Ti_3_C_2_T_*x*_ MXene and LIG substrate (Figure S5d). A sweeping analysis was carried out to reveal the charge storage kinetics based on the peak current (*i*) and the scan rate (*v*) from the CV curves. Assuming *i* obeys a power-law relationship with the *v*:^30^
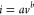
1where *a* and *b* are variable parameters, a straight line was obtained from the plot of log *i vs* log *v*, and the slope equal to exponent *b* (Figure 2c). In two well-defined cases, the *b* value of 0.5 indicates a total diffusion-controlled process, and the *b* value of 1 represents a capacitive-controlled process. For the Ti_3_C_2_T_*x*_/LIG anode, the *b* value was 0.95 and 0.9 (Figure 2c) for the anodic peak and cathodic peak, respectively, indicating a fast redox reaction of proton intercalation of the Ti_3_C_2_T_*x*_/LIG electrode. In a further analysis of the CV curves, the current (*i*) response at a given potential is the sum of two individual charge-storage contributions: surface capacitive contribution and diffusion-controlled contribution.
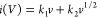
2where *v* represents the scan rate (mV/s) and *k*_1_*v* and *k*_2_*v*^1/2^ are the current response from surface capacitive effects and the diffusion-controlled insertion processes. Eq 2 can be rearranged to eq 3 for analytical purposes:
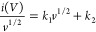
3

By determining the *k*_1_ and *k*_2_ values from the plot of log *i vs* log *v* at different potential points, it is easy to quantify the fraction of the voltammetric current due to the two charge-storage contributions mentioned above. The shaded orange area in Figure 2b represents the capacitive current contribution compared with the total current in the CV curve, which is determined to be 84.7% at a scan rate of 10 mV s^–1^. The capacitive contribution and *b* value for different Ti_3_C_2_T_*x*_ MXene mass loadings are also calculated, as shown in Figure S5a–c. As expected, similar *b* values and contribution percentage results are collected. It should be ascribed to the LIG electrode’s 3D interconnected conductive architecture, which facilitates electron transport and proton diffusion.

Figure 2d shows the galvanostatic charge/discharge (GCD) profiles of the Ti_3_C_2_T_*x*_/LIG electrode collected at the current density from 2 to 100 mA cm^–2^. The triangular-shaped curves show a prominent redox feature around −0.38 V that could be attributed to the predominant proton intercalation redox reactions, which matches the CV analysis very well. Figure 2e shows the specific areal capacitances (calculated based on the geometric dimensions of electrode) of the Ti_3_C_2_T_*x*_/LIG electrode with different mass loadings derived from the GCD curves. Ti_3_C_2_T_*x*_/LIG electrode (0.75 mg cm^–2^) exhibits a specific areal capacitance of 363 mF cm^–2^ at the current density of 2 mA cm^–2^. It drops to 205 mF cm^–2^ at a high current density of 100 mA cm^–2^, with initial capacitance retention of 57%. The specific areal capacitance increased to 839 mF cm^–2^ and 1348 mF cm^–2^ upon increasing the mass loading to 1.8 and 3.2 mg cm^–2^, respectively. The nearly linear correlation reveals that our highly conductive 3D electrode can overcome the insufficient electronic conductivity and electrolyte diffusion in classical electrodes. The Ti_3_C_2_T_*x*_/LIG electrode with high mass loading shows a good rate performance even at a high current density, due to the good ohmic contact between Ti_3_C_2_T_*x*_ nanoflakes and LIG electrode as well as the 3D interconnected nature of the electrode (Figure S6a–c) that facilitates the diffusion of the protons. Furthermore, the long-term cycling test of over 10,000 cycles was conducted using the Ti_3_C_2_T_*x*_/LIG electrode (0.75 mg cm^–2^). Figure 2f shows that 94% of the original capacitance was retained, and nearly 100% Coulombic efficiency was maintained, indicating good cycling stability.

### Positive Electrode Design and Fabrication

**Figure 3:**
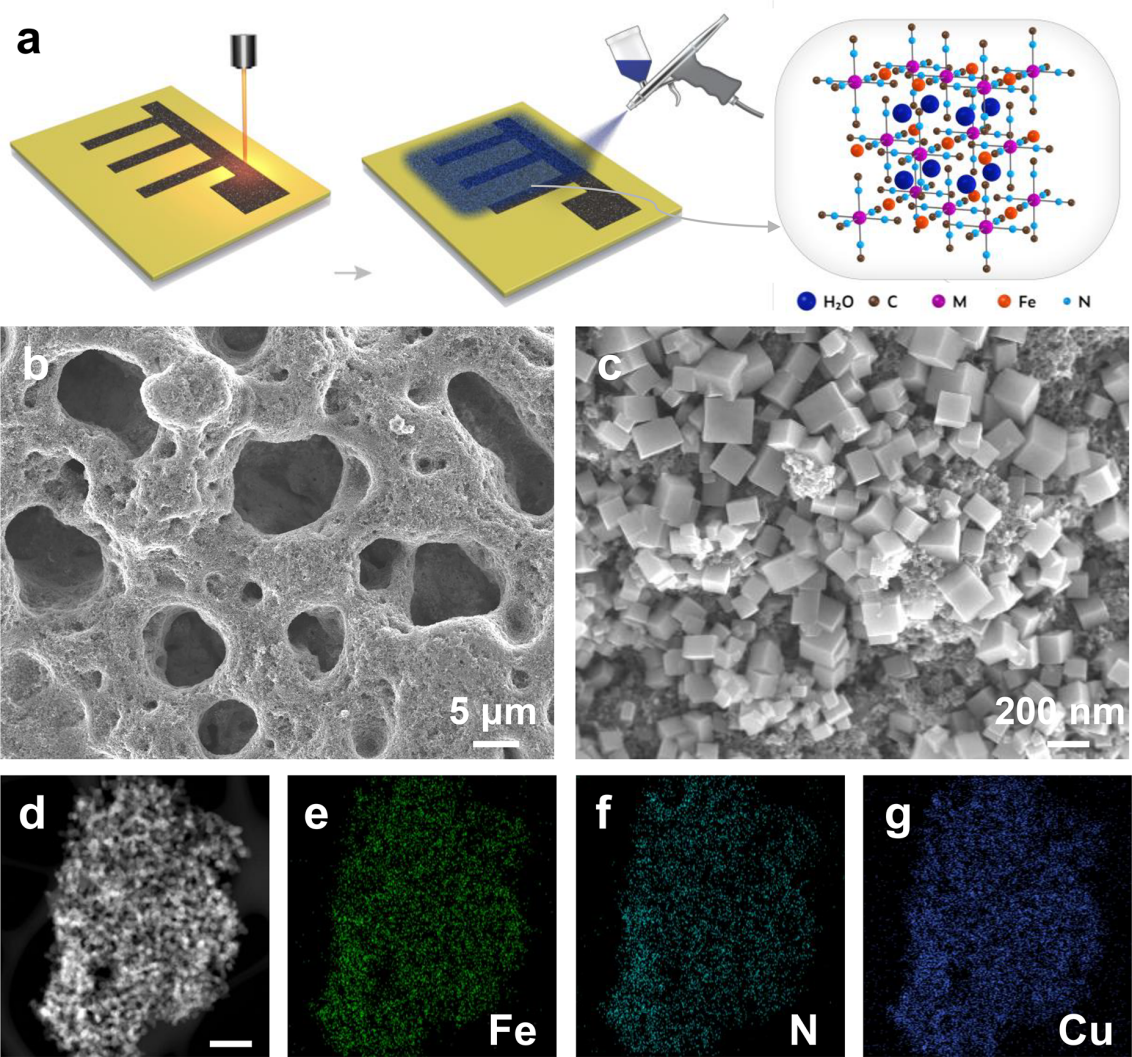
(a) Schematic illustration of the fabrication process of CuFe-PBA/LIG electrode. (b) Low- and (c) high-magnification of SEM images of the CuFe-PBA/LIG electrode. (d) HADDF image of CuFe-PBA nanoparticles (scale bar = 500 nm). (e–g) EDX mapping of Fe, N, and Cu based on the CuFe-PBA nanoparticles.

The fabrication process of the CuFe-PBA/LIG electrode is similar to the Ti_3_C_2_T_*x*_/LIG electrode, as shown in Figure 3a. First, the porous LIG electrode pattern was prepared by the CO_2_ laser (Experimental Section). Then, CuFe-PBA nanoparticles were synthesized by aqueous precipitation with the reported method (see the synthesis details in Experimental Section).^22^ Different characterization results of the CuFe-PBA cathode are given in Figures 3d–g and S4, including X-ray diffraction (XRD), energy dispersive spectroscopy (EDS) for elemental mapping, Fourier transform infrared (FT-IR) spectroscopy, and thermogravimetric analyses (TGA). To prepare a CuFe-PBA based ink, 60 wt % CuFe-PBA, 25 wt % Ketjen black carbon, and 15 wt % Nafion solution were mixed by a tip sonicator to form a homogeneous dispersion (concentration: 4 mg mL^–1^). The Nafion additive works as a binder and proton conductor here: It facilitates the solvation of the ionic groups and accelerates the transfer of protons through mechanisms promoted by the water molecules and hydrogen bonding.^31^ Then, the CuFe-PBA/LIG electrode was fabricated by a spray coating technique on the LIG electrode with the as-prepared ink. Figure 3b shows the SEM image of the CuFe-PBA/LIG electrode. A 3D porous structure is retained after the spray coating process, which may facilitate the electrolyte’s infiltration. The magnified SEM image in Figure 3c shows the homogeneous distribution of CuFe-PBA and carbon black on the surface of the LIG electrode.

**Figure 4:**
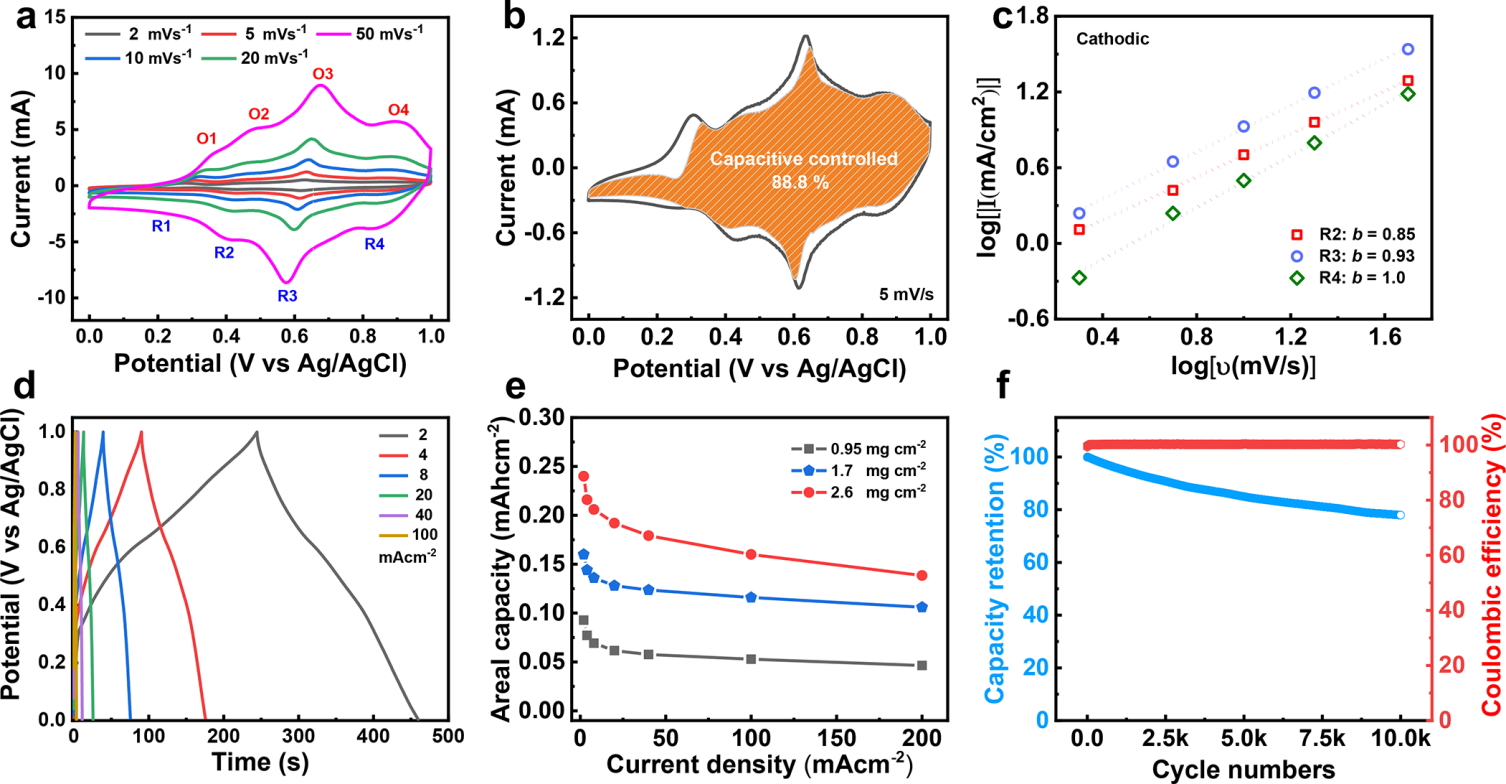
(a) CV curves of CuFe-PBA/LIG electrode at the scan rates of 2–50 mV s^–1^ in the potential range of 0 to 1.0 V (*vs* Ag/AgCl). (b) Deconvolution of charge storage contributions of the CuFe-PBA/LIG electrode (capacitive-controlled *vs* diffusion-controlled currents). (c) The *b* values derived from the CV curves. (d) GCD profiles of the CuFe-PBA/LIG electrode at the current density of 2–100 mA cm^–2^. (e) Areal capacity calculated from the GCD profiles. (f) The cycling performance of the CuFe-PBA/LIG electrode at the fixed current density of 20 mA cm^–2^.

The electrochemical performance of the CuFe-PBA/LIG electrode was evaluated in a three-electrode system in 2 M H_2_SO_4_. CV curves of CuFe-PBA/LIG electrode (CuFe-PBA mass loading of 0.95 mg cm^–2^) were recorded at varied scan rates (Figure 4a), revealing four pairs of redox peaks in the voltage area of 0–1 V (*vs* Ag/AgCl). The O1/R1 redox peaks should be associated with Cu^II^/Cu^I^, and the remaining peaks should be related to Fe^III^/Fe^II^.^32,33^ These redox peaks for Cu^I^/Cu^I^ were not apparent compared with the remaining peaks, attributed to the spontaneous reaction between dissolved oxygen and Cu^I^, which was not recorded by the CV curves. The capacitive current contribution of the CuFe-PBA/LIG electrode was also calculated, as shown in Figure 4b. The shaded orange area represents the capacitive current contribution compared with the total current, estimated to be 88.8% at a scan rate of 10 mV s^–1^. This fast capacitive behavior contributes significantly to the high-rate performance of the CuFe-PBA/LIG electrode. The *b* values (calculated by eq 1) for R_2_, R_3_, and R_4_ were 0.85, 0.93, and 1, respectively (Figure 4c), suggesting the fast pseudocapacitive redox reaction between Fe^III^ and Fe^II^. The collective resistive contributions from the CuFe-PBA/LIG electrode and the electrolyte were 3.2 Ω, as calculated from the Nyquist impedance plot (Figure S7).

The GCD curves (Figure 4d) of the CuFe-PBA/LIG electrode were recorded at the current density from 2 to 100 mA cm^–2^ (mass loading of 0.95 mg cm^–2^). The deviation of the triangular-shaped curves could be associated with the redox reactions of the Fe^III^/Fe^II^ couple, which agrees with the CV analysis. The electrochemical performance of the CuFe-PBA/LIG electrode can still perform well upon increasing the mass loading (Figure 4e), which is related to the 3D architecture of the CuFe-PBA/LIG electrode (Figure S8). The specific areal capacity increased from 0.093 mAh cm^–2^ to 0.16 mAh cm^–2^ and 0.24 mAh cm^–2^ with the mass loading of 1.7 mg cm^–2^ and 2.6 mg cm^–2^. Furthermore, the CuFe-PBA/LIG electrode shows decent long-term cycling stability, retaining 78% of initial capacity after 10,000 cycles along with a high Coulombic efficiency close to 100% (Figure 4f).

### Symmetric Microsupercapacitors

**Figure 5:**
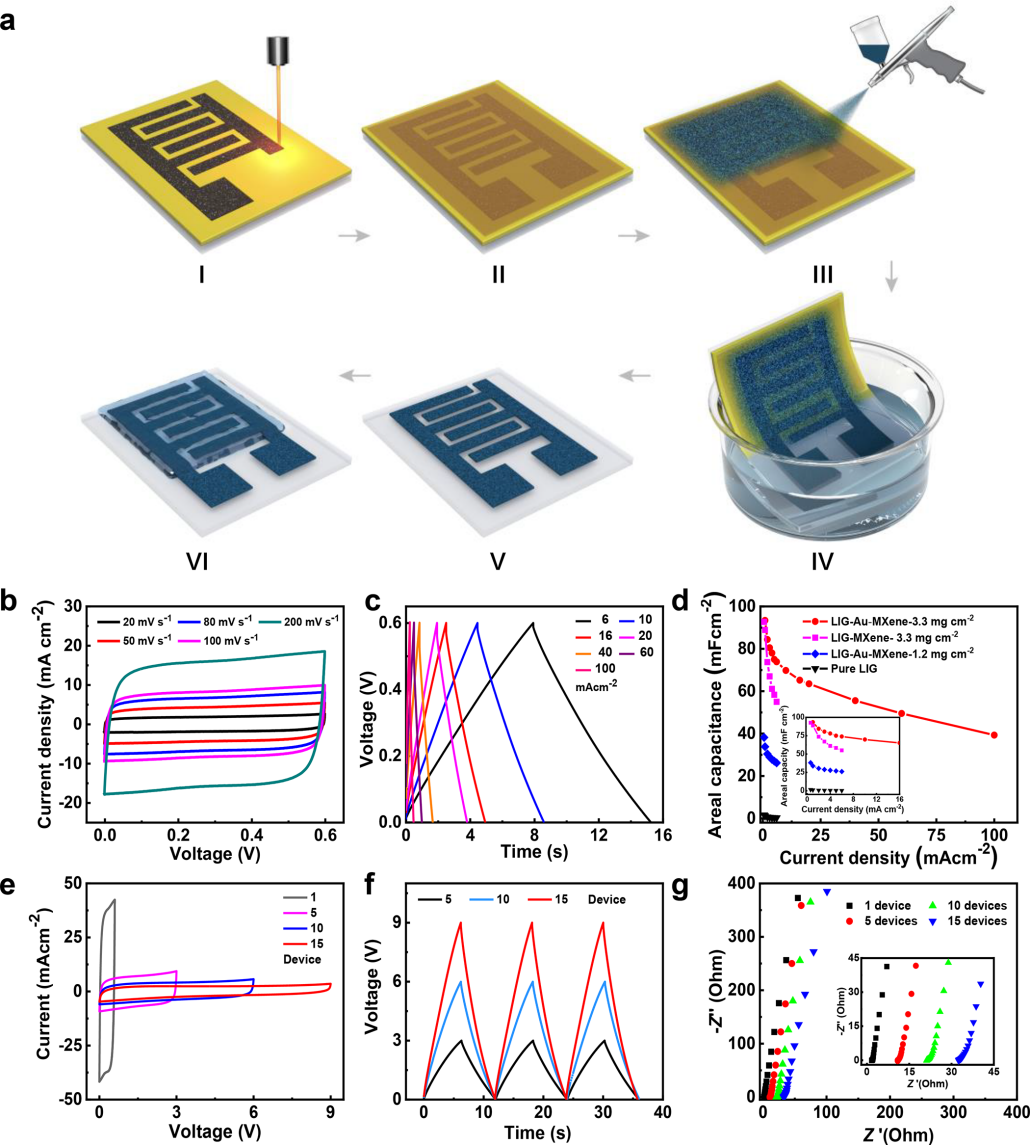
Electrochemical performance of the symmetric Ti_3_C_2_T_*x*_ based MSC. (a) Schematic illustration of the fabrication process of MSCs. (b) CV curves of MSC at the scan rates of 20–200 mV s^–1^ in the voltage window of 0 to 0.6 V. (c) GCD profiles of MSC at the current density of 6 to 100 mA cm^–2^. (d) Areal specific capacity calculated from the GCD profiles, compared with the other MSCs with different fabrication conditions. (e) CV curves of the assembled MSCs with the various device numbers. (f) GCD profiles of the assembled MSC with the various device numbers. (g) Nyquist impedance plot for the assembled MSC with the different device numbers; insert shows the zoom-in profiles.

After evaluating the performance of the separate electrode, a Ti_3_C_2_T_*x*_ MXene-based symmetric MSCs (mass loading 3.3 mg cm^–2^) was first prepared following the schematic fabrication process (Figure 5a). First, 3D porous LIG electrode patterns were printed by CO_2_ laser (Figure 5a, step I). An Au layer was deposited by sputter coating to enhance the electrode’s conductivity (Figure 5a, step II, Figure S15). Then, the small flakes of Ti_3_C_2_T_*x*_ were sprayed onto the electrode directly. After that, the electrode pattern was immersed in water to remove the unexposed parts (Figure 5a, steps IV and V), as the PVA/lignin composite is water soluble. Finally, a PVA/H_2_SO_4_ gel electrolyte was coated on the electrode (Figure 5a, step VI). As seen in Figure 5b, typical quasi-rectangular CV curves were recorded with a voltage window of 0.6 V, indicating the capacitive behavior. Furthermore, the MSCs endured a high scan rate CV test (to 8000 mV s^–1^) while showing acceptable capacitance attenuation (Figure S9a). The capacitive current contribution of the MSC was calculated according to eq 3, estimated to be 86.5% with a scan rate of 50 mV s^–1^(Figure S9c), which also proves the capacitive-dominated behavior. Charge–discharge curves in Figures 5c and S9b show a typical equilateral triangle shape, which agrees with the CV results. The MSC device delivers a specific areal capacitance of 93 mF cm^–2^ at a current density of 2 mA cm^–2^ and maintains 40 mF cm^–2^ at a high current density of 100 mA cm^–2^ (Figure 5d). For comparison, the symmetric capacitor was prepared without the Au layer at the same mass loading level, and the capacitance drops dramatically as the current increases (inset of Figure 5d). Such a high-rate performance of the MSCs should be attributed to the 3D conductive network of the LIG electrode and the fast redox kinetics of the Ti_3_C_2_T_*x*_ MXene. Furthermore, the MSC device shows good cycling stability, retaining 89% of the original capacitance after 10,000 cycles along with a high Coulombic efficiency close to 100% (Figure S9d).

Despite the narrow voltage window of a single symmetric Ti_3_C_2_T_*x*_-based MSC, the mask-free and spray coating strategies make it straightforward to prepare the integrated MSCs array with high voltage. Thus, integrated MSCs with a 9 V voltage window were designed to demonstrate this idea, as shown in Figure S10. As expected, all the CV curves of a single unit, 5 units, 10 units, and 15 units connected in series exhibited quasi-rectangular shapes, suggesting the typical EDLC behavior (Figure 5e). The integrated MSCs arrays exhibited a specific areal energy density of 45.3 μWh cm^–2^ at a power density of 28.4 mW cm^–2^. The outstanding tandem capacitive behaviors were also illustrated by the charge–discharge profiles (Figure 5f), which show symmetrical triangle shapes and a stable charge–discharge time, indicating the uniformity of a single MSC device. Furthermore, the series-connected capacitive behaviors were confirmed by EIS. As shown in Figure 5g, the Nyquist plot exhibits a nearly vertical straight line in the low-frequency area, suggesting a predominant capacitive behavior and fast ionic diffusion.^34^ The equivalent series resistance (ESR) was 1.9, 10.8, 21.2, and 32.5 Ω, respectively, roughly proportional to the increased number of device units. Therefore, our strategy is capable of preparing integrated MSCs arrays with adjustable electrochemical performance.

### Hybrid Microsupercapacitors

**Figure 6:**
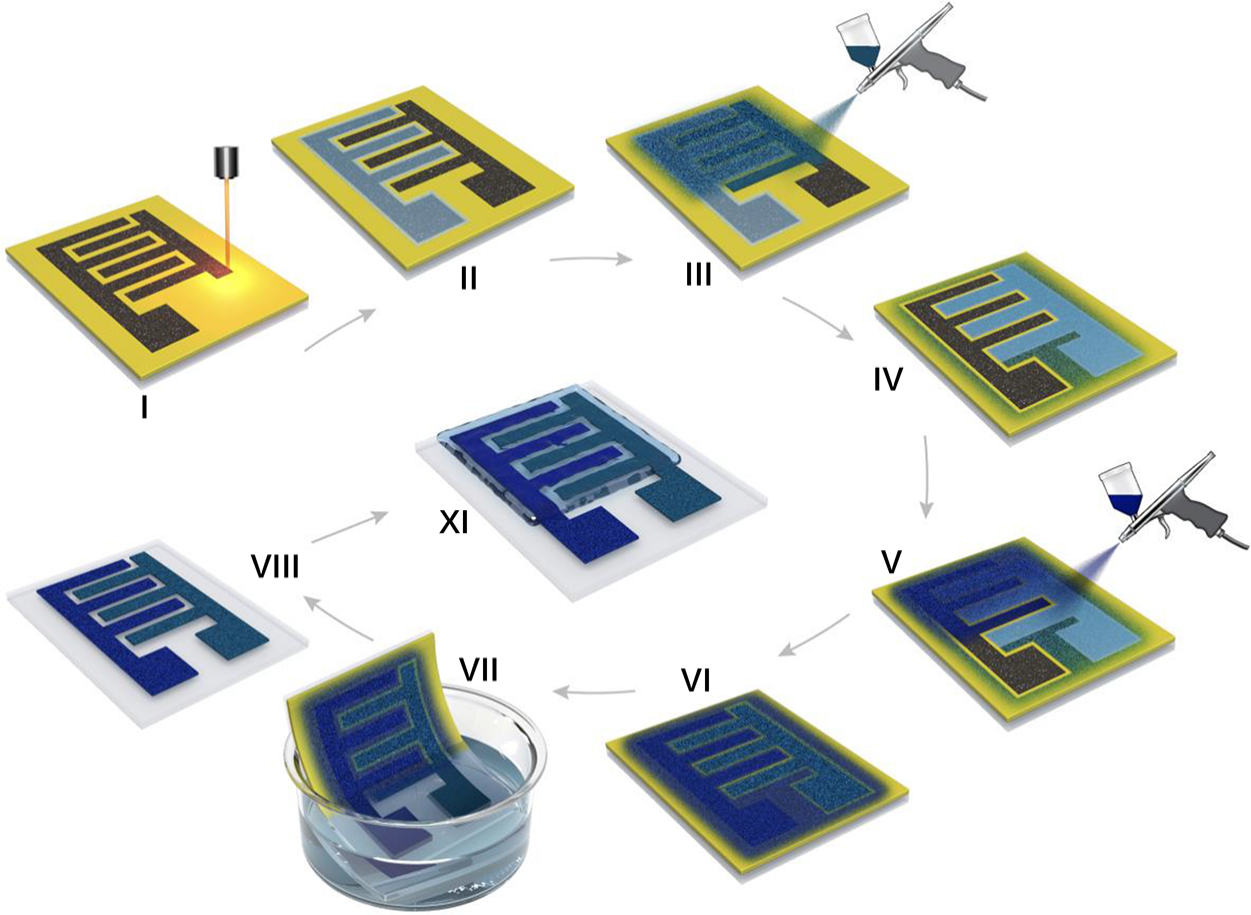
Schematic illustration of the fabrication process of hybrid Ti_3_C_2_T_*x*_/CuFe-PBA microsupercapacitor. Step I: The porous LIG electrode pattern was prepared by the CO_2_ laser. Step II: A polyimide mask covered one electrode in advance. Steps III–VI: The Ti_3_C_2_T_*x*_ flakes and CuFe-PBA were sprayed onto the electrodes separately. Step VII: The unexposed parts were removed by the water lift-off process. Step VIII: PVA/H_2_SO_4_ gel electrolyte was used as the electrolyte.

**Figure 7:**
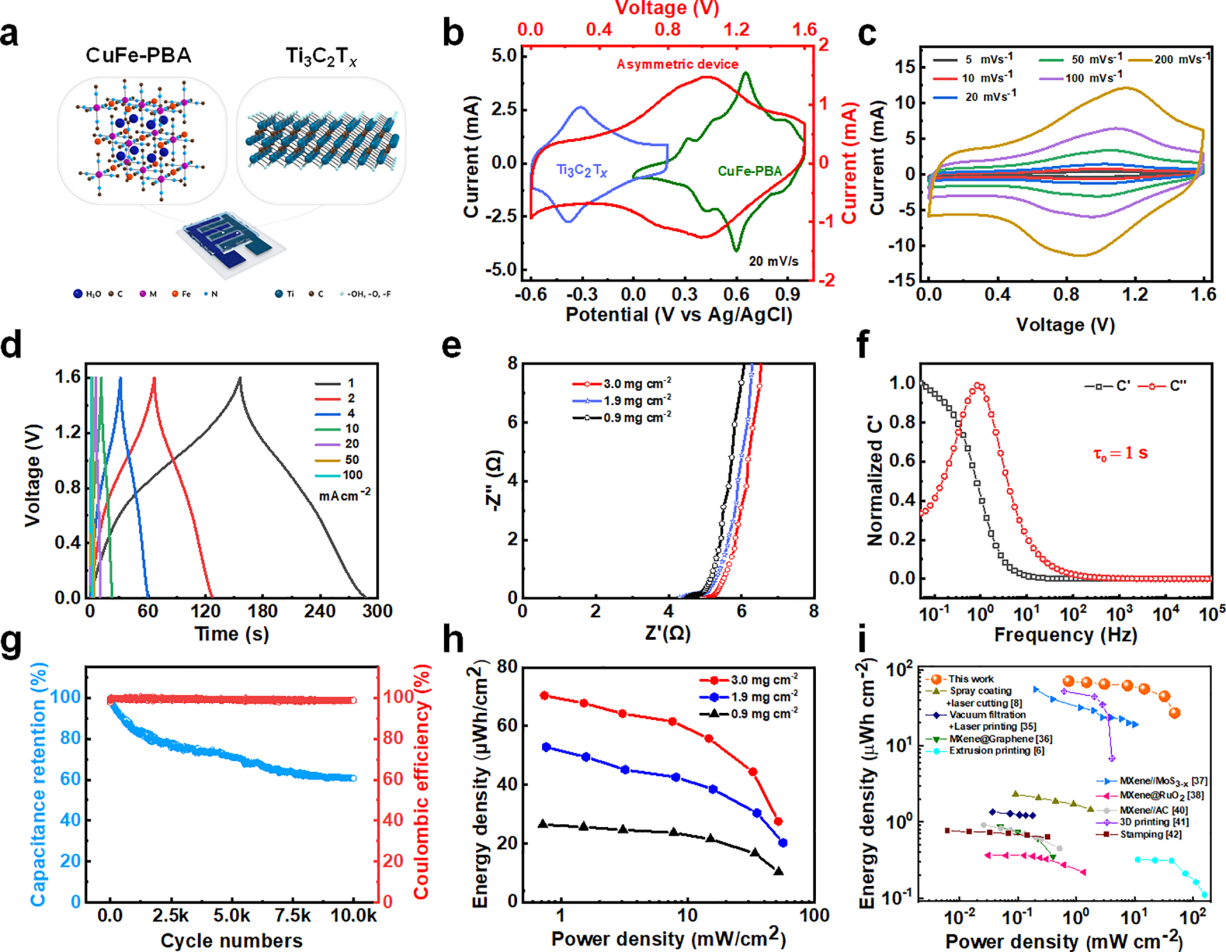
Electrochemical performance of the Ti_3_C_2_T_*x*_/CuFe-PBA hybrid microcapacitors. (a) Illustration of the hybrid microcapacitor. (b) CV curves of Ti_3_C_2_T_*x*_/LIG, CuFe-PBA/LIG, and hybrid microcapacitor at a scan rate of 20 mV s^–1^. (c) CV curves of the hybrid capacitor at scan rates of 5–200 mV s^–1^ in the voltage window of 0 to 1.6 V. (d) GCD profiles of the hybrid microcapacitor at the current density of 1–100 mA cm^–2^. (e) Nyquist impedance plot for the hybrid microcapacitors with different mass loadings. (f) Normalized imaginary capacitance (*C*′′) and real capacitance (*C*′) *vs* frequency for the hybrid microcapacitor. (g) The cycling performance of the hybrid microcapacitor at the fixed current density of 20 mA cm^–2^. (h) Ragone plot showing the energy and power density of the hybrid microcapacitors with different mass loadings. (i) Ragone plot for this work in comparison to other state-of-the-art Ti_3_C_2_T_*x*_ MXene-based microcapacitors.

Finally, a scalable microfabrication process using lignin-based laser lithography and a water lift-off method was applied to fabricate the asymmetric H-MSCs according to the schematic fabrication process (Figures 6, 7a, and S1). First, the porous LIG electrode pattern was prepared by the CO_2_ laser. Second, a polyimide mask covered one electrode in advance (Figure 6, step II). With the help of the mask, the Ti_3_C_2_T_*x*_ flakes and CuFe-PBA were sprayed onto the electrodes separately (Figure 6, steps III–VI). The unexposed parts were also removed *via* the water lift-off process (Video S1). Finally, an acidic gel electrolyte (PVA/H_2_SO_4_) was used. To optimize the capacity of CuFe-PBA/LIG and Ti_3_C_2_T_*x*_/LIG electrodes, the mass loading of each electrode was balanced by eq S2, and the corresponding charge balance chart of Ti_3_C_2_T_*x*_ and CuFe-PBA electrodes is shown in Table S1. The geometric dimensions of the assembled asymmetric H-MSC device are shown in Figure S11. Figure 7b displays the CV curves of CuFe-PBA/LIG, Ti_3_C_2_T_*x*_/LIG, and the asymmetric H-MSC device, which shows an operating voltage of 1.6 V (total active materials mass loading 0.9 mg cm^–2^). CV curves of the asymmetric device at different scan rates were recorded in Figure 7c, where two prominent redox peaks at 0.98 and 1.02 V were observed, indicating reversible faradaic redox reactions. The capacitive current contribution of the H-MSC device was calculated according to eq 3, estimated to be 84.5% with a scan rate of 20 mV s^–1^ (Figure S13), which confirms the capacitive-dominated behavior.

GCD profiles of hybrid capacitors in Figure 7d were recorded at the current density of 1–100 mA cm^–2^, showing a nearly triangular shape with apparent redox curvature around 0.9 V, in agreement with the CVs. The H-MSCs deliver an areal capacity of 74.6 mF cm^–2^ at the current density of 1 mA cm^–2^. The device maintains at 30.5 mF cm^–2^ when the current density increases to 100 mA cm^–2^, showing a good rate performance. This high-rate performance of our H-MSCs device should be attributed to the high conductivity and 3D porous architecture of the LIG electrode that favors proton diffusion. The ESR values from the Nyquist plot were estimated to be 4.5–5 Ω for different mass loadings (Figure 7e). The ESR values hardly change when increasing the mass loading. The low resistance in our device is likely related to the highly conductive framework of the 3D LIG. Moreover, the outstanding rate performance of the asymmetric hybrid device is validated by the short characteristic relaxation time constant τ_0_ (1/*f*_0_, *f*_0_ where *C*′′ is maximum) of 1 s (Figure 7f). Finally, the H-MSC device endured long-term cycling (over 10,000 cycles), and it retains 62% of the original capacity, showing acceptable capacitance attenuation as an H-MSC device (Figure 7g).

An H-MSC device with a mass loading of 3 mg cm^–2^ was also fabricated, delivering an areal capacitance of 198 mF cm^–2^ at the current density of 1 mA cm^–2^; further, a 75.8 mF cm^–2^ was retained even at a practical/high current density to 100 mA cm^–2^, indicating good rate performance (Figure S12). The as-prepared H-MSC device shows a high areal energy density of 70.5 μWh cm^–2^ at a power density of 0.74 mW cm^–2^ and 27.6 μWh cm^–2^ at a high-power density of 52 mW cm^–2^ (Figure 7h). Moreover, the capacitances of the asymmetric devices are 74.5, 148, and 198 mF cm^–2^ with mass loading increases from 0.9, 1.9, to 3 mg cm^–2^ at a current density of 1 mA cm^–2^, assuming nearly linear interpolation between the two. And the linear correlation stays (capacitances increase from 46, 85.5, to 125 mF cm^–2^ with mass loading increase from 0.9, 1.9, to 3 mg cm^–2^) even at the ultrahigh current density (50 mA cm^–2^), which shows a good rate performance. The linear correlation between capacitances of the asymmetric devices and active materials mass loading should be attributed to the high conductivity of Ti_3_C_2_T_*x*_ MXene and the 3D interconnected architecture of the LIG electrode, which facilitates proton diffusion. The performance drop at the ultrahigh current density of 100 mA cm^–2^ was found only with the mass loading of 3 mg cm^–2^, which was expected, as electron transport and ionic diffusion in the H-MSCs device will be hindered within the high mass loading electrode. These metrics are significantly higher than those recently reported MXene-based microsupercapacitors. Figure 7i compares the areal energy and power density of our device with different previously reported MXene-based microcapacitors.^6,8,35−42^ Our H-MSCs asymmetric device shows a better electrochemical performance than other MXene-based pseudocapacitive microsupercapacitors thanks to the fast Faradaic reactions in the CuFe-PBA electrode, suggesting high capability and cost-effective production of microsupercapacitor devices.

## Conclusions

In summary, we have developed hybrid microsupercapacitors using Ti_3_C_2_T_*x*_ MXene and CuFe-PBA electrodes and a proton-based electrolyte. A scalable microfabrication process using lignin-based laser-induced graphene electrodes and a water lift-off lithography method was applied to fabricate the hybrid microsupercapacitors. The hybrid microsupercapacitors offer a maximum energy density (70.5 μWh cm^–2^) and power density (52 mW cm^–2^) with good rate performance. The proposed process is scalable and compatible with existing microfabrication technologies, which can be used for highly integrated micropower units for on-chip energy storage applications.

## Experimental Section

### Materials and Chemicals

Lithium fluoride (LiF, ≥98%), hydrochloride acid (HCl, 35–37%), sulfuric acid (H_2_SO_4_, 98%), poly(vinyl alcohol) (PVA, *M*_w_ = 98000), copper(II) sulfate (CuSO_4_, ≥98%), potassium ferricyanide (K_3_FeCN_6_, ≥96%) were purchased from Sigma-Aldrich Chemicals company and used without purification. The Ti_3_AlC_2_ power (98%, 400 mesh) was purchased from Carbon-Ukraine company.

### Preparation of Ti_3_C_2_T_*x*_ MXene Dispersion

Ti_3_C_2_T_*x*_ nanoflakes were prepared by selectively etching an Al interlayer from Ti_3_AlC_2_ with an *in situ* HF-forming etchant. Typically, 1 g of LiF was added to 20 mL of 9 M HCl under stirring. One g of Ti_3_AlC_2_ powder was slowly added to the etchant under stirring, then transferred to an oil bath at 35 °C and stirred for 24 h. The suspension was washed with deionized (DI) water until the pH reached ≥6 *via* centrifugation at 3500 rpm to remove the salts and acid. After the pH reached 6, Ti_3_C_2_T_*x*_ nanoflakes were collected after 30 min of centrifugation at 4000 rpm. The small flakes Ti_3_C_2_T_*x*_ ink were prepared by a tip sonicator in a cold bath for 40 min (40% of the full power, with 4 s on and 4 times off).

### Preparation of CuFe-PBA Dispersion

The CuFe-PBA was prepared by an aqueous precipitation strategy as previously reported. Typically, 20 mL of CuSO_4_ solution (0.2 M) was added into 20 mL of K_3_FeCN_6_ (0.1 M) solution by drop under vigorous stirring. After 6 h, the precipitate was washed with DI water three times and collected *via* centrifugation at 10000 rpm. CuFe-PBA based ink was prepared by mixing the CuFe-PBA, conductive carbon black, and Nafion solution (the ratio by weight: 65%: 25%: 10%) and redispersed with the help of a tip sonicator.

### Preparation of Lignin-Based LIG Electrode

The LIG electrodes were prepared by a lignin laser lithography method.^43^ First, 5 g of lignin and 5 g of PVA were mixed in 50 mL of DI water and stirred in a 70 °C oil bath for 10 h. The thin film was prepared by the blade coating method. The electrode pattern was fabricated *via* a direct CO_2_ laser-scribing process (10.6 μm, 75 W, Universal PLS6,75, Universal Laser Systems Inc., AZ, USA). The applied laser power was set at 5% of the full power with a laser pulse of 1000 pulses per inch, a scan rate of 3%, and a *z* distance of 3 mm.

### Preparation of Hybrid Microdevices

First, the designed LIG electrode patterns were prepared by the laser-scribing process. Then the as-prepared Ti_3_C_2_T_*x*_ and CuFe-PBA-based ink were used for spraying coating *via* an airbrush (Anest Iwata, Japan) with instantaneous drying using a hot air gun. The mass loading of the active materials was controlled by the volume of the ink used. The unexposed parts were removed by the water lift-off process, leaving the transformed electrode on the surface of the substrate (Video S1). Then, the H_2_SO_4_-based gel electrolyte was prepared by mixing H_2_SO_4_ (2g) with 10 mL of DI water and PVA (1 g) at 80 °C for 1 h. The obtained H_2_SO_4_/PVA gel was dropped on the top of the Ti_3_C_2_T_*x*_ and CuFe-PBA electrodes.

### Characterization

TEM tests and SAED were conducted by Titan 80–300 ST, FEI. SEM images were conducted by Merlin, Zeiss, Germany. Raman spectroscopy measurements were performed using a micro-Raman spectrometer (LabRAM ARAMIS, Horiba-Jobin Yvon). XRD patterns were recorded by a Bruker diffractometer (D8 Advance) with Cu Kα radiation (λ= 0.15406 nm). AFM image was collected by Bruker Instruments Dimension Icon. FT-IR spectra were recorded in the range of 700–4000 cm^–1^ by a Nicolet 6700 spectrometer. TGA was conducted using a Netzsch TG 209 within the temperature range from 25 to 700 °C at a heating rate of 5 °C per minute.

### Electrochemical Measurements and Calculation

All the electrochemical measurements were conducted on a Biologic VMP3 workstation. The standard three-electrode system was used to evaluate the electrochemical performance of the electrodes, in which Ag/AgCl was used as the reference electrode, active carbon film as the counter electrode, and 2 M H_2_SO_4_ solution as the electrolyte.

The specific areal capacitance derived from GCD curves was calculated using the following equation:
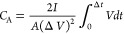
4where *I* (A) is the constant current, *A* (cm^2^) is the active electrode area, Δ*t* (s) is the period upon the discharge process, and Δ*V* is the potential change calculated according to the maximum voltage upon discharge.

The specific areal energy density *E*_A_ and the specific areal power density *P*_A_ were calculated *via* the following equations: The specific areal energy density (μWh cm^–2^):
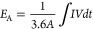
5The specific areal power density (mWh cm^–2^):
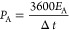
6

## Supporting Information Available

TEM images, SEM images, EIS spectra, TGA pattern, XRD patterns, Raman spectra, FTIR spectra of LIG electrode, Ti_3_C_2_T_*x*_ MXene and CuFe-PBA samples; photographs of the PVA/lignin film, Ti_3_C_2_T_*x*_ MXene solution, MSC device, and H-MSC device; electrochemical performance figures of Ti_3_C_2_T_*x*_/LIG electrode, CuFe-PBA/LIG electrode, MSC device, and H-MSC device (PDF)
Video S1: The water lift-off process of the Ti_3_C_2_T_*x*_/CuFe-PBA hybrid microsupercapacitors (MP4)
