# Supplementary material for: Three-Dimensional Ti3C2Tx MXene-Prussian Blue Hybrid Microsupercapacitors by Water Lift-Off Lithography
Source: ACS Nano. 2022 Jan 28;16(2):1974–85. doi: 10.1021/acsnano.1c06552 (PMC8867912; doi:10.1021/acsnano.1c06552)
Supplement: Supplementary file 1 — nn1c06552_si_001.pdf [file nn1c06552_si_001.pdf]

Three-Dimensional $Ti_3C_2T_x$ MXene-Prussian Blue Hybrid Microsupercapacitors by Water Lift-Off Lithography

Yongjiu Lei[1†], Wenli Zhao[2†], Yunpei Zhu[1], Ulrich Buttner[3], Xiaochen Dong[2], Husam N. Alshareef[1,]*

Dr. Y. J. Lei, Dr. W. L. Zhao, Dr. Y. P. Zhu, Prof. H. N. Alshareef

[1] Physical Science and Engineering Division, King Abdullah University of Science and Technology (KAUST), Thuwal 23955-6900, Saudi Arabia.

Dr. W. L. Zhao, Prof. X. C. Dong

[2] School of Physical and Mathematical Sciences, Nanjing Tech University, Nanjing 211816, China.

U. Buttner

[3] Nanofabrication Core Lab, King Abdullah University of Science and Technology (KAUST), Thuwal 23955-6900, Saudi Arabia.

*E-mail: husam.alshareef@kaust.edu.sa

Charge balance of hybrid microcapacitors:

The total charge stored in each electrode ($Q_{\text{electrode}}$) is determined by the specific capacitance ($C_{\text{electrode}}$), the active mass (m), and the potential window (ΔE) of each electrode. It can be estimated according to following equation:

$$Q_{electrode} = C_{electrode} \times m \times \Delta E \quad (1)$$

To achieve the charge balance $Q^{+}_{electrode} = Q^{-}_{electrode}$, a mass balance following the equation:

$$\frac{m_+}{m_-} = \frac{C^{-}_{electrode} \times \Delta E_-}{C^{+}_{electrode} \times \Delta E_+} = \frac{C^{-}_{electrode} \times \Delta E_-}{C^{+}_{capacity}} \quad (2)$$

Where $C^{-}_{electrode}$ is the gravimetric specific capacitance of the negative electrode, and $C^{+}_{capacity}$ is the gravimetric specific capacity of the positive electrode. Thus, the optimal mass ratio between the two electrodes m_+/m_- can be adjusted to get the optimal performance of an asymmetric supercapacitor. The areas of both electrodes are maintained as the same. For the asymmetric device, mass loadings of MXene and CuFe-PBA were controlled by the volumes of CuFe-PBA and $Ti_3C_2T_x$ solutions used in the spray coating process and optimized as shown in Table S1.

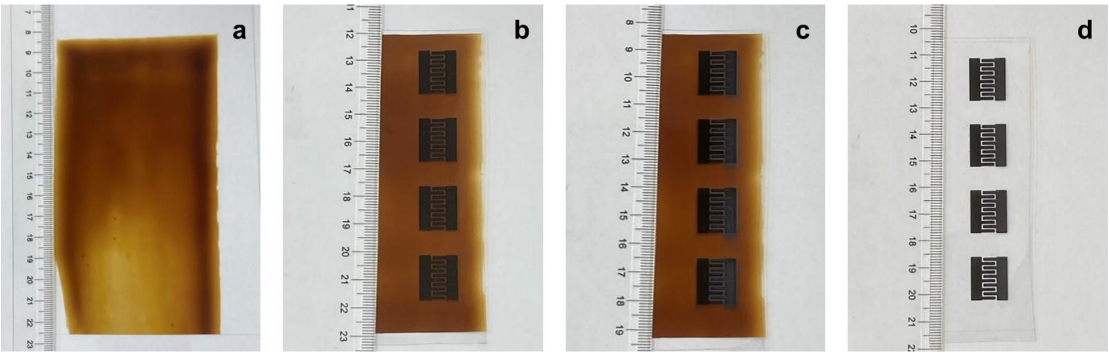

Figure S1. The photographs of the a) PVA/lignin film, b) lignin-derived LIG patterns, c) MXene/CuFe-PBA/LIG device prepared by a spray coating method, d) MXene/CuFe-PBA/LIG device after water-lift off process (the unit of the scale bars is centimeter).

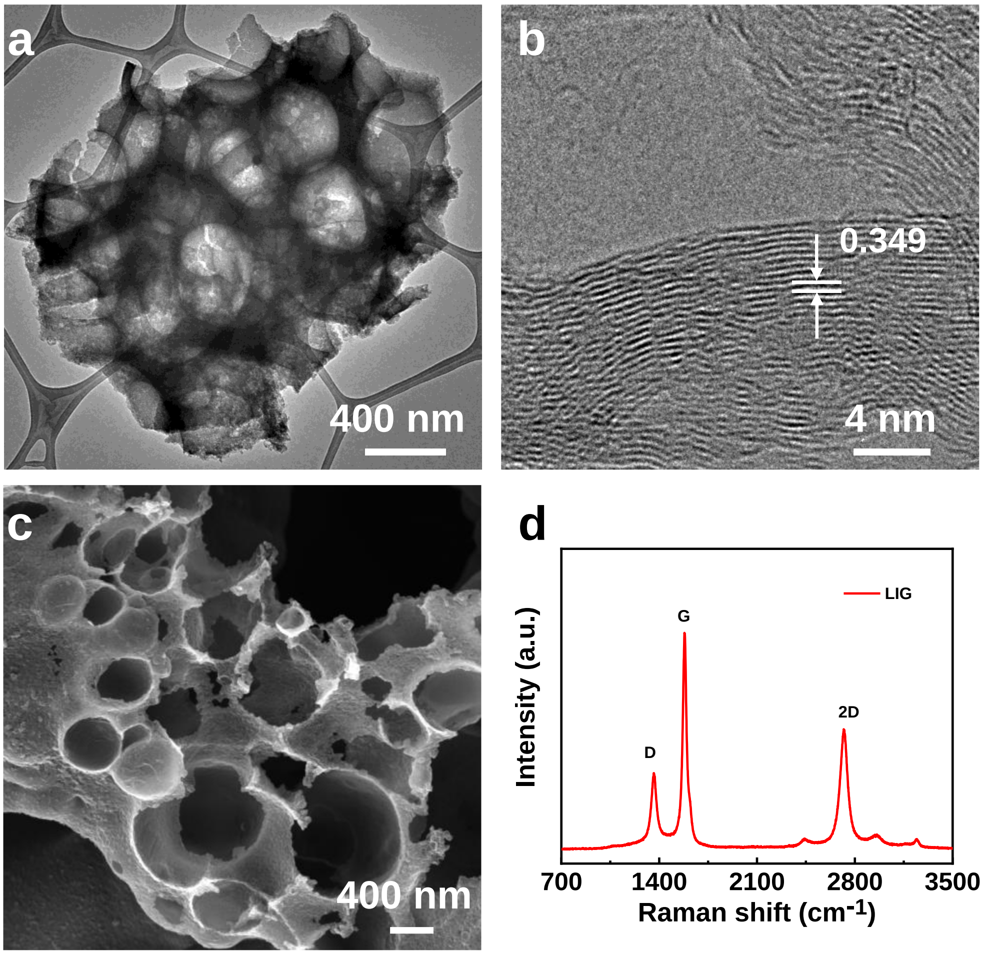

Figure S2. a) The transmission electron microscopy (TEM) image of LIG. b) High resolution TEM image of LIG. c) High resolution SEM image of LIG. d) The Roman spectra of the lignin derived LIG.

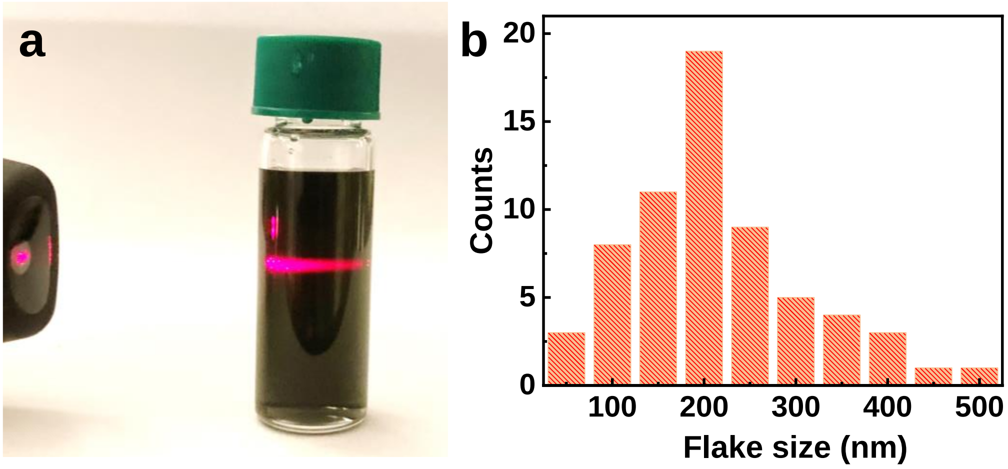

Figure S3. a) the Tyndall scattering effect of $Ti_3C_2T_x$ MXene solution. b) the $Ti_3C_2T_x$ flake size distribution measured by AFM.

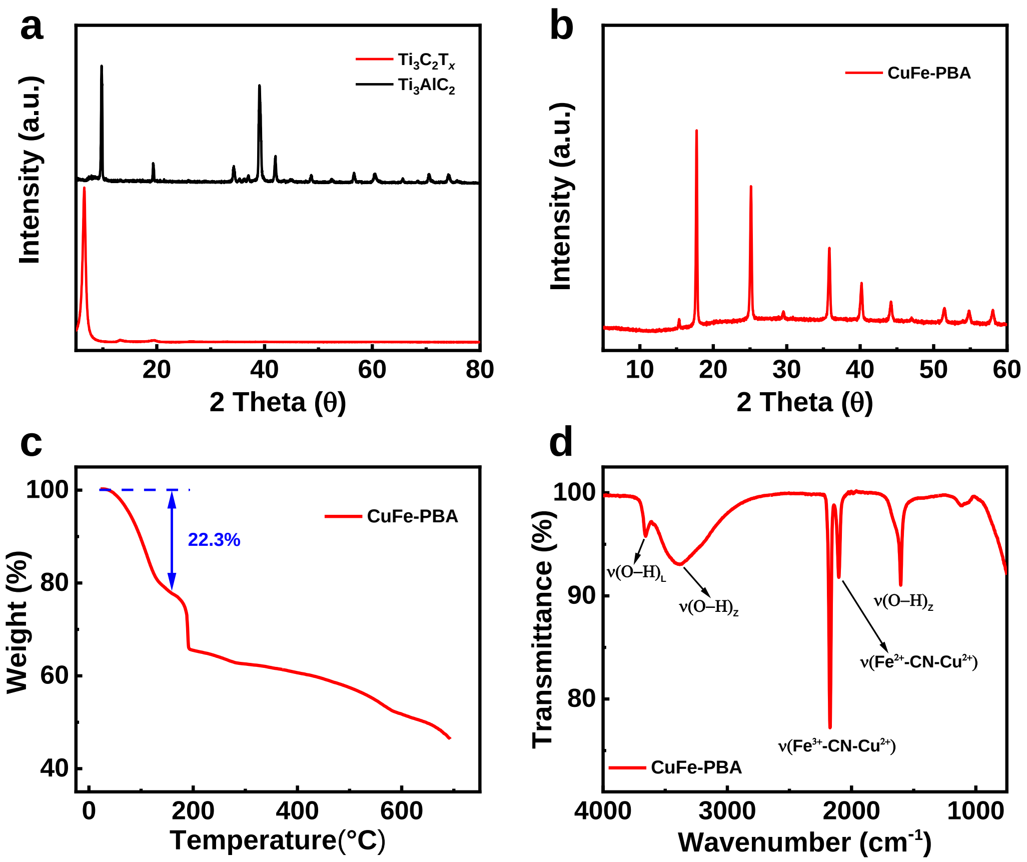

Figure S4. a) XRD patterns of delaminated $Ti_3C_2T_x$ films and Ti_3AlC_2 powder. b) XRD pattern of the synthesized CuFe-PBA powder. c) Thermogravimetric analyses of the synthesized CuFe-PBA, the sample was tested under N_2 atmosphere at a heating rate of 5 °C/min, and the result matches the previously reported data.[1] d) Fourier transform infrared spectroscopy (FTIR) of the CuFe-PBA sample, the broad peak at ~3300 cm^{-1} and sharp peak at ~3700 cm^{-1} are attributed to the vibration of zeolitic water and ligand water, respectively.[2]

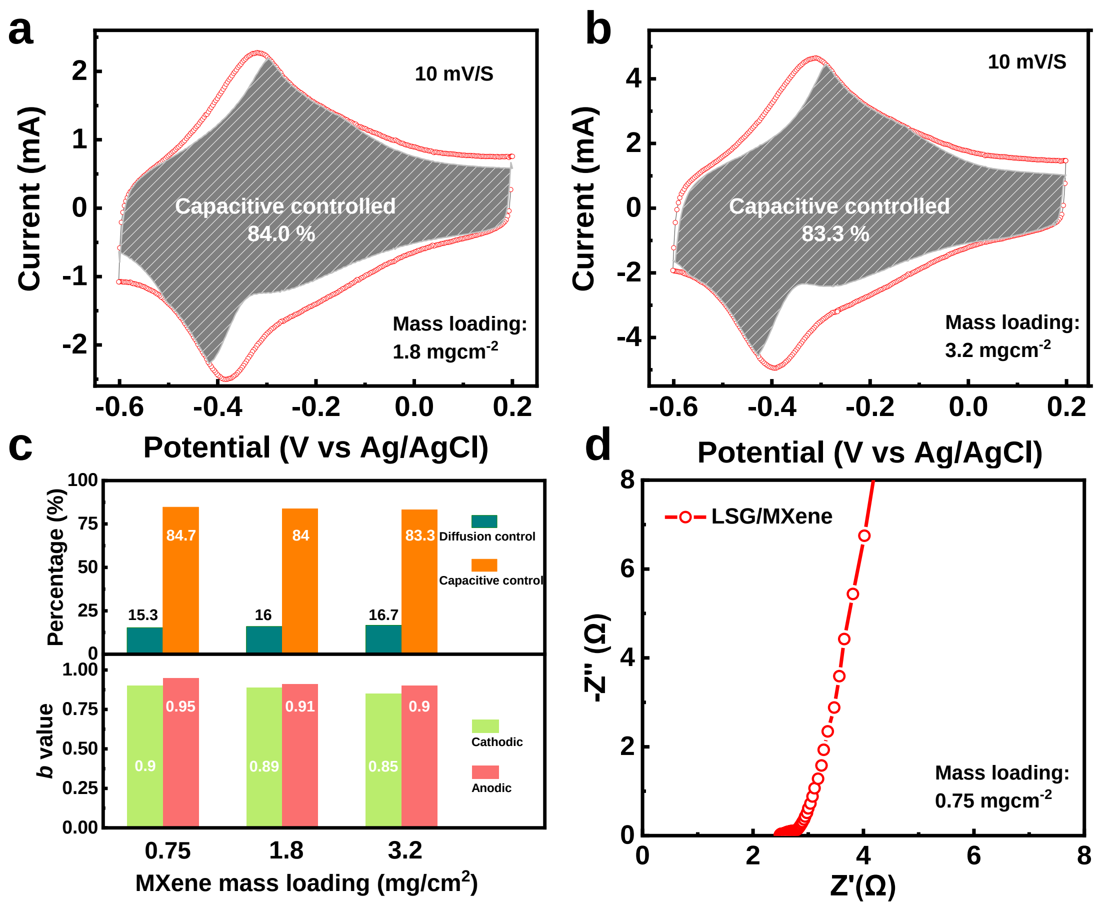

Figure S5. a,b) Deconvolution of charge storage contributions of the $Ti_3C_2T_x$/LIG electrodes with different mass loading. c) Capacitive-controlled *vs.* diffusion-controlled, and the *b* values derived from the CV curves of $Ti_3C_2T_x$/LIG electrodes with different mass loading. d) Nyquist impedance plot for the $Ti_3C_2T_x$/LIG electrode.

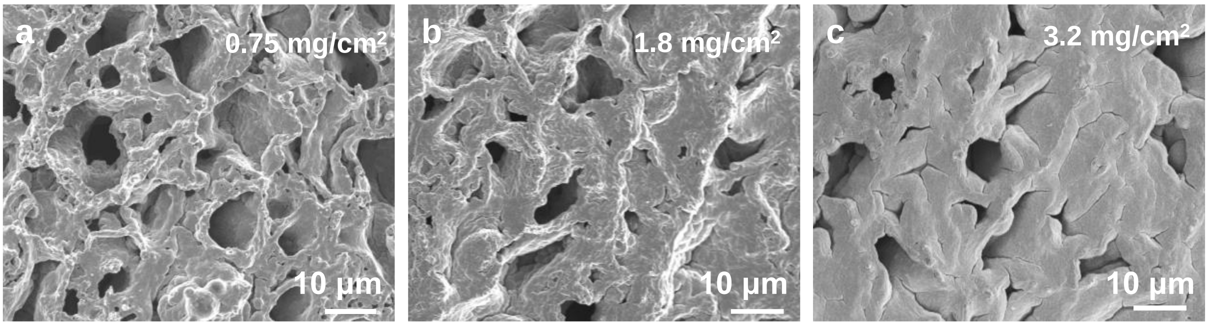

Figure S6. a-c) The SEM images of $Ti_3C_2T_x$/LIG electrodes with different mass loading.

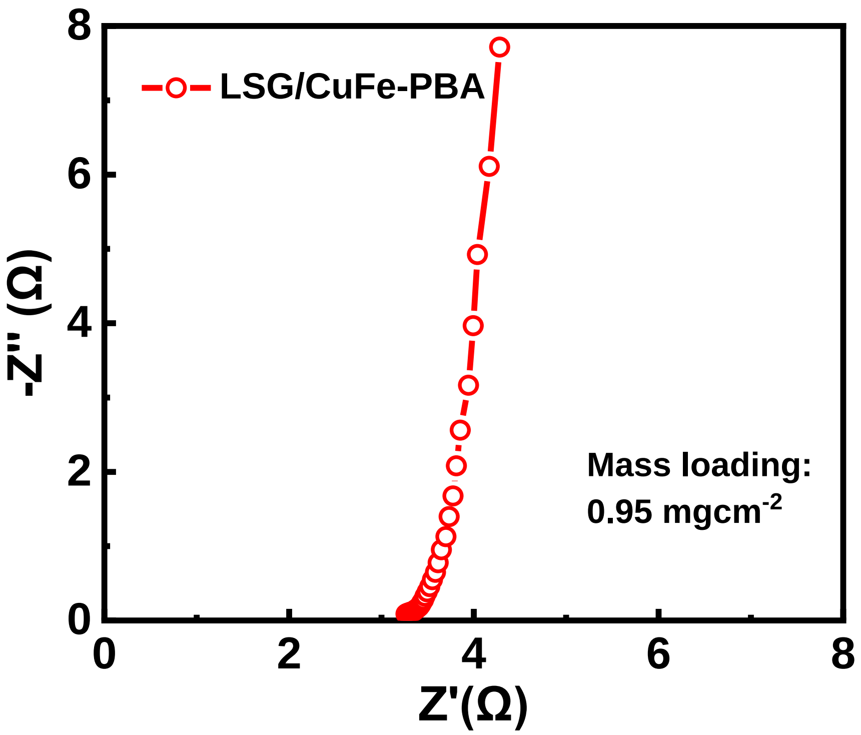

Figure S7. Nyquist impedance plot for the CuFe-PBA/LIG electrode.

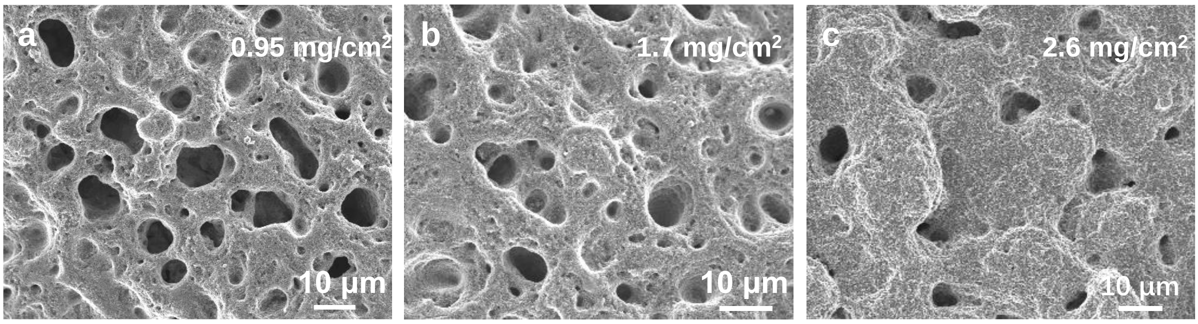

Figure S8. a-c) The SEM images of CuFe-PBA/LIG electrodes with different mass loading.

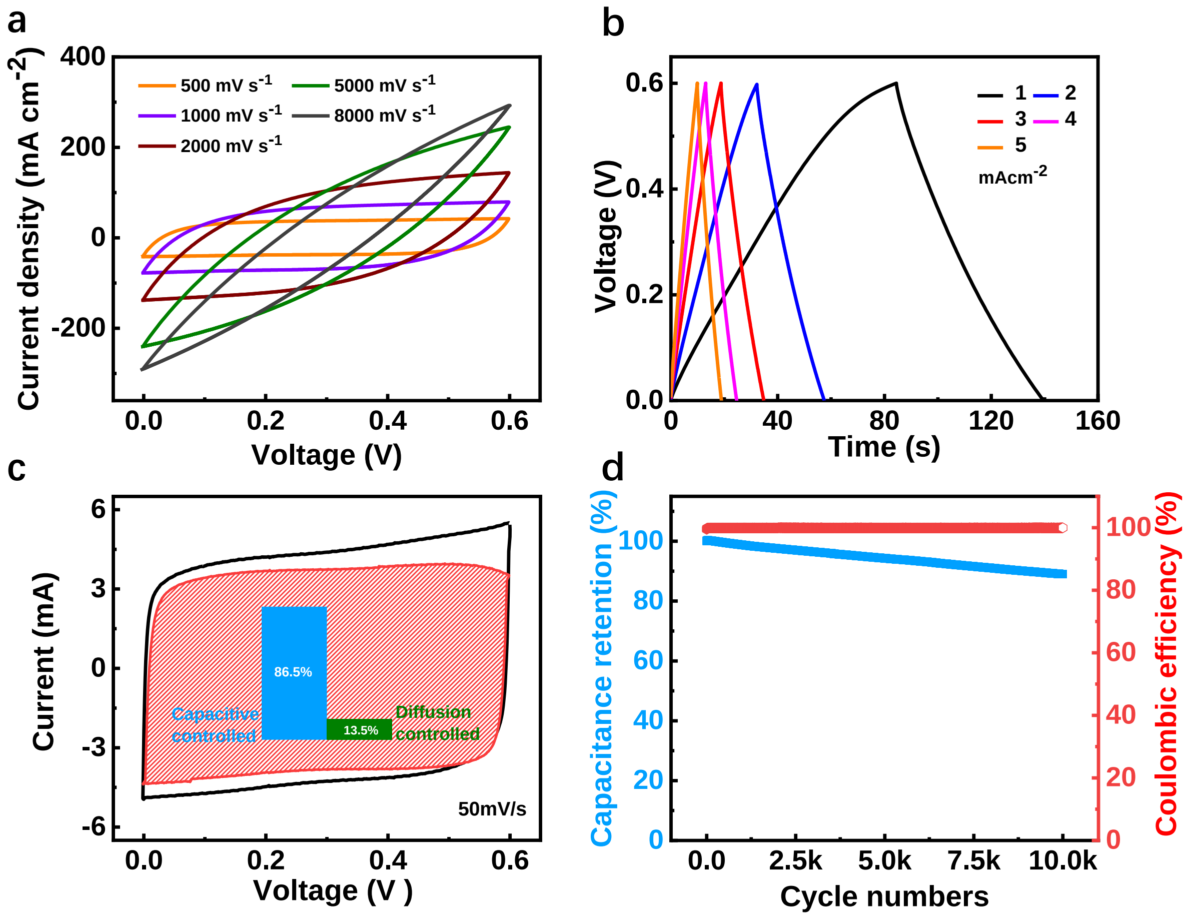

Figure S9. a) CV curves of MSC at the scan rates of 500 - 2000 mV s^{-1} in the voltage window of 0 to 0.6 V. b) GCD profiles of MSC at the current density of 1 to 5 mA cm^{-2}. c) Deconvolution of charge storage contributions of the MSC device (capacitive-controlled *vs.* diffusion-controlled). d) The cycling performance of the MSC device at the fixed current density of 20 mA cm^{-2}.

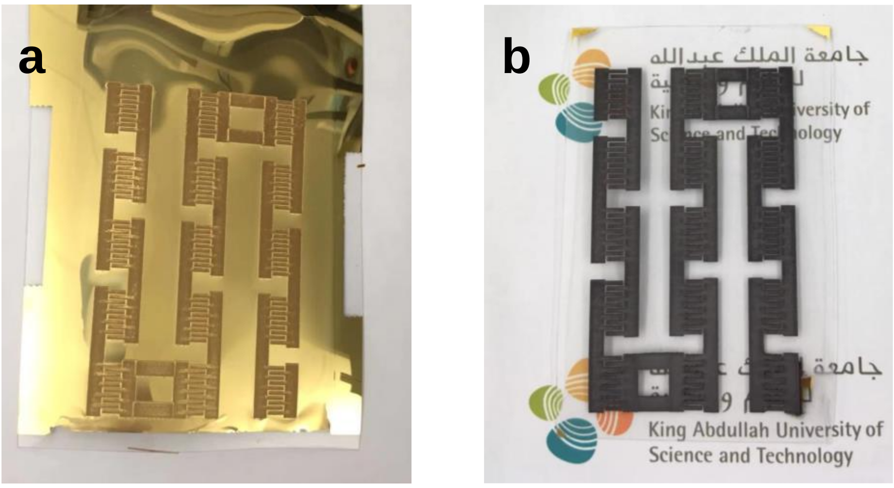

Figure S10. The photographs of a) fifteen Au/LIG electrodes, b) fifteen symmetric $Ti_3C_2T_x$/Au/LIG based MSCs after the water lift-off process.

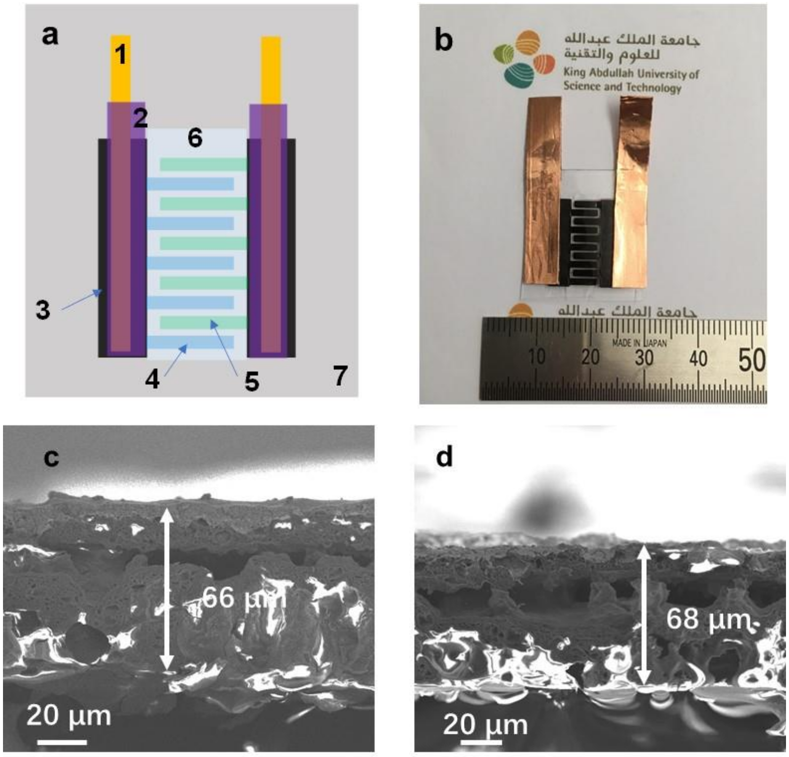

Figure S11. a) The geometric dimensions of the assembled asymmetric H-MSC device, the dimension of the LIG finger electrode is 5 mm *1 mm, and the space between the fingers is 400 µm; (1) is the copper strip electrode, (2) is polydimethylsiloxane(PDMS) polymer that protects the copper from the PVA/H_2SO_4 gel, (3) is the LIG substrate, (4) is the CuFe-PBA on LIG finger electrode, (5) is the $Ti_3C_2T_x$ on LIG finger electrode, (6) is the PVA/H_2SO_4 gel electrolyte, (7) is the plastic substrate. b) The photograph of the assembled asymmetric H-MSC device. The side view SEM images of c) CuFe-PBA/LIG electrode, and d) $Ti_3C_2T_x$/LIG electrode.

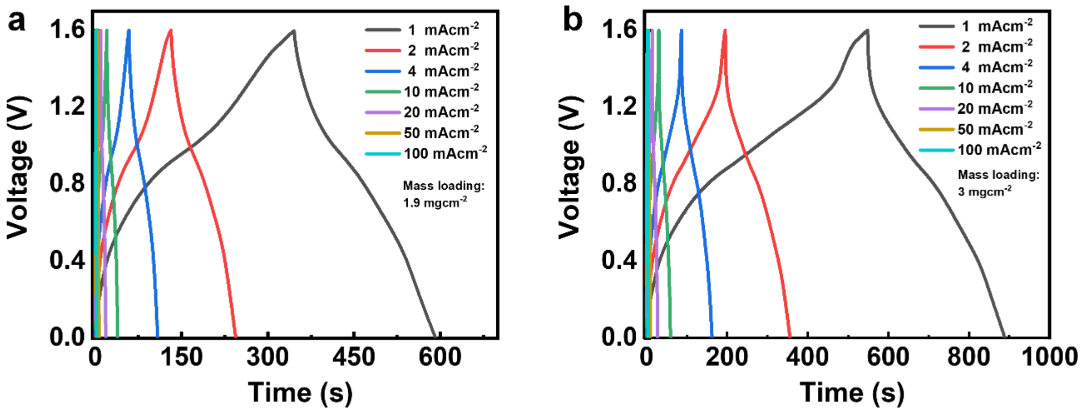

Figure S12. a, b) GCD profiles of the asymmetric H-MSCs with different mass loading at the current density of 1 to 100 mA cm^{-2}.

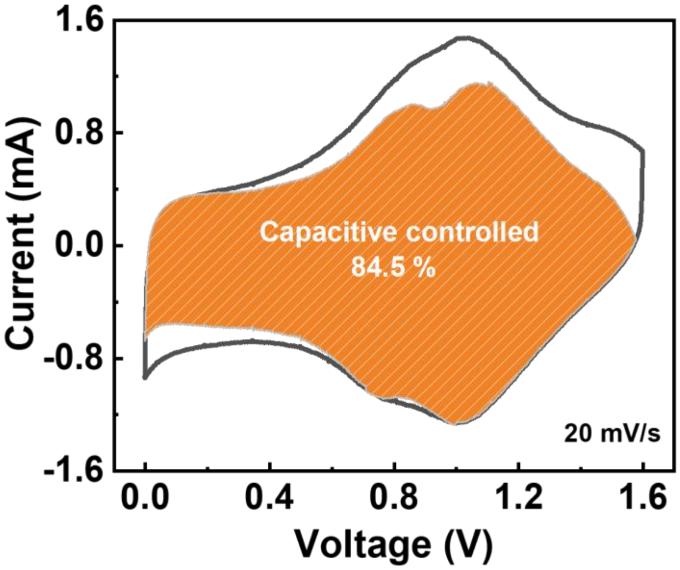

Figure S13. Deconvolution of charge storage contributions of the asymmetric H-MSC.

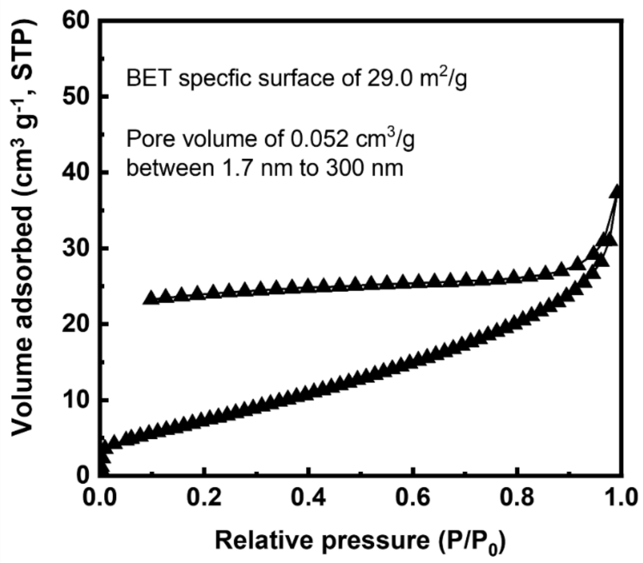

Figure S14. The average adsorption–desorption isotherm of N_2 at 77 K of the porous LIG electrode materials.

The isotherm curve of LIG material shows a type IV isotherm with the hysteresis loop of N_2. The observed specific surface area was 29.0 m^2/g, and the average volume has been calculated to be 0.052 cm^3/g. This result reveals that the LIG electrode is a microporous material.

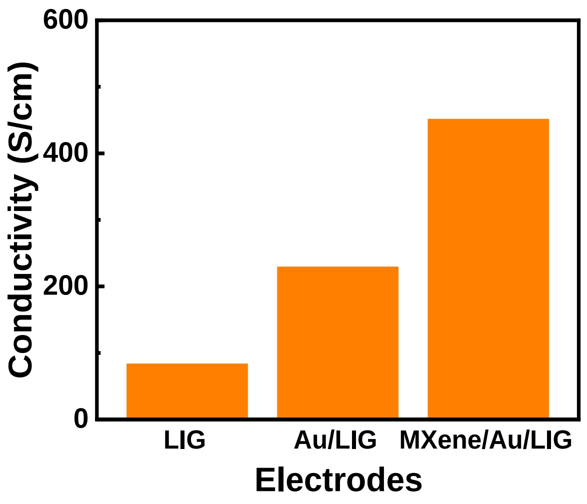

Figure S15. The conductivities of LIG, Au/LIG and MXene/Au/LIG electrodes.

The conductivity of lignin-induced graphene (LIG) is 83.3 S cm^{-1}, which is higher than the reported polyimide-induced graphene (25 S cm^{-1})[3], and even higher than those of widely utilized conductive Ketjen black (31.6 S cm^{-1}) and super P (46.5 S cm^{-1})[4]. We have compared the conductivity of LIG, Au/LIG, and MXene/Au/LIG electrodes. A gold layer deposited by sputter coating can further enhance the electrode's conductivity, which is crucial for the high-power density of the microsupercapacitors.

Table S1. Charge balance of $Ti_3C_2T_x$ and CuFe-PBA electrodes

Mass loading of Device (mg/cm^2)	Mass loading of MXene/LIG (mg/cm^2)	Mass loading of CuFe-PBA/LIG (mg/cm^2)	Charge of MXene/LIG electrode C_- ($Coulomb/cm^2$)	Charge of CuFe-PBA/LIG electrode C_+($Coulomb/cm^2$)	Ratio (C_-/C_+)
0.9	0.83	0.98	0.32	0.34	0.94
1.9	1.7	2.1	0.63	0.7	0.9
3.0	2.83	3.15	0.95	1.04	0.91

REFERENCES

(1) Wu, X.; Hong, J. J.; Shin, W.; Ma, L.; Liu, T.; Bi, X.; Yuan, Y.; Qi, Y.; T. Surta, T. W.; Huang, W.; Neuefeind, J.; Wu, T.; P. Greaney, A.; Jun Lu, J.; Ji, X. Diffusion-free Grotthuss topochemistry for high-rate and long-life proton batteries. *Nat. Energy*, **2019**, *4*, 594−603.

(2) Kulesza, P. J.; Malik, M. A.; Denca, A.; Strojek, J. In situ FT-IR/ATR spectroelectrochemistry of Prussian blue in the solid state. *Anal. Chem*. **1996**, *68*, 2442−2446.

(3) Lin, J.; Peng, Z.; Liu, Y.; Ye, R.; Samuel, E. L.; Ruiz-Zepeda, F.; Yacaman, M. J.; Yakobson, B. I.; Tour, J. M. Laser-Induced Porous Graphene Films from Commercial Polymers. *Nat. Commun.* **2014**, *5*, 5714.

(4) Mao, M.; Wang, S.; Lin, Z.; Liu, T.; Hu, Y. S.; Li, H.; Huang, X.; Chen, L.; Suo, L. Electronic Conductive Inorganic Cathodes Promising High‐Energy Organic Batteries. *Adv. Mater.* **2021**, *33*, 2005781.
